# Incidence and outcomes of perioperative myocardial infarction/injury diagnosed by high-sensitivity cardiac troponin I

**DOI:** 10.1007/s00392-021-01827-w

**Published:** 2021-03-25

**Authors:** Danielle M. Gualandro, Christian Puelacher, Giovanna Lurati Buse, Noemi Glarner, Francisco A. Cardozo, Ronja Vogt, Reka Hidvegi, Celia Strunz, Daniel Bolliger, Johanna Gueckel, Pai C. Yu, Marcel Liffert, Ketina Arslani, Alexandra Prepoudis, Daniela Calderaro, Angelika Hammerer-Lercher, Andreas Lampart, Luzius A. Steiner, Stefan Schären, Christoph Kindler, Lorenz Guerke, Stefan Osswald, P. J. Devereaux, Bruno Caramelli, Christian Mueller, for the BASEL-PMI Investigators, Stella Marbot, Ivo Strebel, Alessandro Genini, Katharina Rentsch, Jasper Boeddinghaus, Thomas Nestelberger, Karin Wild, Tobias Zimmermann, Alberto J. S. Duarte, Andreas Buser, Nelson de Luccia, Luca Koechlin, Desiree Wussler, Joan Walter, Velina Widmer, Michael Freese, Pedro Lopez-Ayala, Raphael Twerenbold, Patrick Badertscher, Esther Seeberger, Thomas Wolff, Edin Mujagic, Arne Mehrkens, Julia Dinort, Gregor Fahrni, Raban Jeger, Christoph Kaiser, Mariana Matheus, Adriana F. Pastana

**Affiliations:** 1 Department of Cardiology and Cardiovascular Research Institute Basel (CRIB), University Hospital Basel, University of Basel, Spitalstrasse 2, CH-4056 Basel, Switzerland; 2 Interdisciplinary Medicine in Cardiology Unit, Cardiology Department, Heart Institute (InCor), University of Sao Paulo Medical School, São Paulo, Brazil; 3 Department of Anesthesiology, University Hospital Düsseldorf, Düsseldorf, Germany; 4 Laboratory Medicine, Heart Institute (InCor), University of Sao Paulo Medical School, São Paulo, Brazil; 5 Department of Anesthesiology, University Hospital Basel, University of Basel, Basel, Switzerland; 6 Department of Laboratory Medicine, Cantonal Hospital Aarau, Aarau, Switzerland; 7 Department of Laboratory Medicine, University of Basel, Basel, Switzerland; 8 Department of Clinical Research, University of Basel, Basel, Switzerland; 9 Department of Spinal Surgery, University Hospital Basel, Basel, Switzerland; 10 Department of Anesthesiology, Cantonal Hospital Aarau, Aarau, Switzerland; 11 Department of Vascular Surgery, University Hospital Basel, University of Basel, Basel, Switzerland; 12 Population Health Research Institute, David Braley Cardiac, Vascular and Stroke Research Institute, Anesthesiology, Perioperative Medicine, and Surgical Research Unit C/o Hamilton General Hospital, McMaster University, Hamilton, Canada

**Keywords:** Myocardial infarction, Myocardial injury, Non-cardiac surgery, High-sensitivity troponin, Perioperative care

## Abstract

**Background:**

Perioperative myocardial infarction/injury (PMI) diagnosed by high-sensitivity troponin (hs-cTn) T is frequent and a prognostically important complication of non-cardiac surgery. We aimed to evaluate the incidence and outcome of PMI diagnosed using hs-cTnI, and compare it to PMI diagnosed using hs-cTnT.

**Methods:**

We prospectively included 2455 patients at high cardiovascular risk undergoing 3111 non-cardiac surgeries, for whom hs-cTnI and hs-cTnT concentrations were measured before surgery and on postoperative days 1 and 2. PMI was defined as a composite of perioperative myocardial infarction (PMI_Infarct_) and perioperative myocardial injury (PMI_Injury_), according to the Fourth Universal Definition of Myocardial Infarction. All-cause mortality was the primary endpoint.

**Results:**

Using hs-cTnI, the incidence of overall PMI was 9% (95% confidence interval [CI] 8–10%), including PMI_Infarct_ 2.6% (95% CI 2.0–3.2) and PMI_Injury_ 6.1% (95% CI 5.3–6.9%), which was lower versus using hs-cTnT: overall PMI 15% (95% CI 14–16%), PMI_Infarct_ 3.7% (95% CI 3.0–4.4) and PMI_Injury_ 11.3% (95% CI 10.2–12.4%). All-cause mortality occurred in 52 (2%) patients within 30 days and 217 (9%) within 1 year. Using hs-cTnI, both PMI_Infarct_ and PMI_Injury_ were independent predictors of 30-day all-cause mortality (adjusted hazard ratio [aHR] 2.5 [95% CI 1.1–6.0], and aHR 2.8 [95% CI 1.4–5.5], respectively) and, 1-year all-cause mortality (aHR 2.0 [95% CI 1.2–3.3], and aHR 1.8 [95% CI 1.2–2.7], respectively). Overall, the prognostic impact of PMI diagnosed by hs-cTnI was comparable to the prognostic impact of PMI using hs-cTnT.

**Conclusions:**

Using hs-cTnI, PMI is less common versus using hs-cTnT. Using hs-cTnI, both PMI_Infarct_ and PMI_Injury_ remain independent predictors of 30-day and 1-year mortality.

**Graphic abstract:**

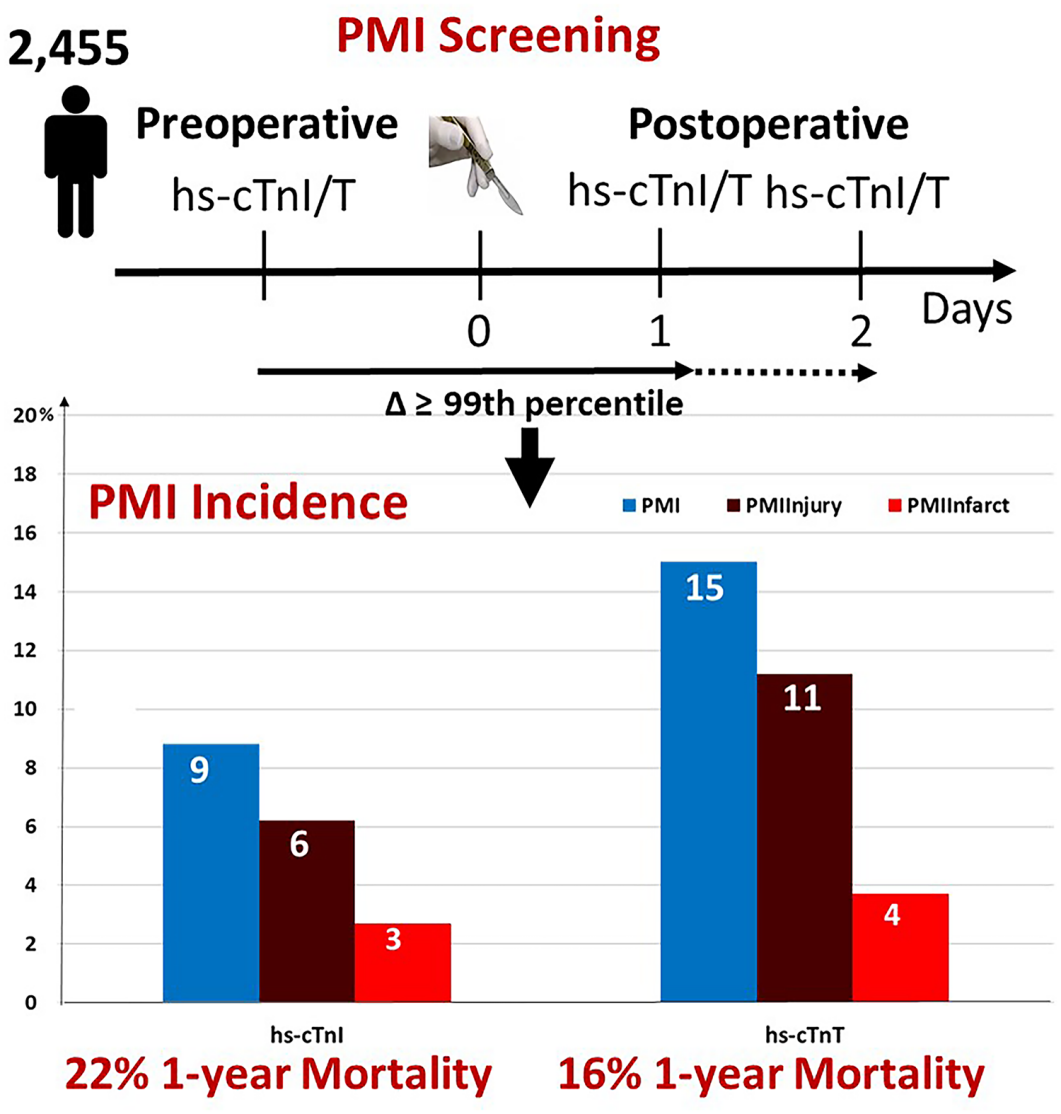

**Supplementary Information:**

The online version contains supplementary material available at 10.1007/s00392-021-01827-w.

## Introduction

More than 300 million surgical interventions are performed annually worldwide [1]. Despite improvements in surgical techniques and anesthesia, the rate of postoperative mortality remains a substantial population problem.[2, 3] Cardiac complications including perioperative myocardial infarction/injury (PMI) have recently been identified as causal contributors to a substantial number of these deaths [4, 5]. Moreover, PMI portends substantial risk of major cardiovascular events in the subsequent year.

The detection of cardiac complications following non-cardiac surgery is challenging for several reasons. [6, 7] Symptoms in this setting are often unspecific or even absent due to analgesia. In addition, clinical signs have low sensitivity, patients are usually not seen by cardiologists, and cardiac imaging and/or cardiac biomarkers are not routinely included in the postoperative care [4, 5, 8, 9]. A study implementing routine screening for PMI using preoperative and postoperative measurements of high-sensitivity cardiac troponin (hs-cTn) T found that PMI is asymptomatic in 85% of patients [4]. The same applies to patients with myocardial injury after noncardiac surgery (MINS) considered due to coronary artery disease (CAD). [5, 10, 11] Accordingly, active surveillance is essential for detecting perioperative cardiac complications. [4–7, 12]

While the incidence and outcome of PMI and MINS diagnosed using preoperative and postoperative measurements of hs-cTnT have recently been determined, it is currently unknown whether these findings also apply when using hs-cTnI. [4, 5, 10, 11] This is of major concern, as cTnI is used more frequently worldwide than cTnT. In addition, evidence for possibly clinically relevant pathophysiological and analytical differences has recently emerged between cTnI and cTnT. For example, cTnT concentrations, but not cTnI, exhibit a diurnal rhythm [13]. In addition, cTnT concentrations have a stronger association with renal dysfunction, which is common in the perioperative setting, than cTnI. [14, 15]

Therefore, the aim of our study was to evaluate the incidence and outcome of PMI and MINS diagnosed by hs-cTnI, and to directly compare it to PMI and MINS diagnosed with hs-cTnT.

## Methods

### Patients

BASEL-PMI is an ongoing diagnostic study accompanying a systematic PMI screening and response program in high-risk patients with routine measurements of perioperative cTn concentrations [4]. Briefly, since 2014, consecutive patients undergoing non-cardiac surgery at the University Hospital Basel and the Cantonal Hospital Aarau, both in Switzerland, as well as consecutive patients scheduled for arterial vascular surgery at the Heart Institute, University of Sao Paulo Medical School, Brazil, have been enrolled [4]. The inclusion criteria are age 65–85 years or age above 45 years in the presence of established coronary artery disease, peripheral artery disease or cerebrovascular disease. Patients whose surgery had been cancelled and those who had had cardiac surgery in the two weeks preceding the operation were excluded. For the main analysis, we included consecutive patients in whom at least two measurements of hs-cTnI and hs-cTnT concentrations were available simultaneously. For analyses addressing 30-day and 1-year mortality/major adverse cardiovascular events (MACE), each patient was included only once at first enrollment.

The local ethics committees approved the protocol (NCT02573532/CAPPESQ 610,608), and all patients provided written consent. We adhered to the STROBE guidelines for observational studies (Supplemental eTable 1).

### Perioperative assessment

Before surgery, cardiac risk was classified based on the Revised Cardiac Risk Index (RCRI)[16], and surgical risk was classified as proposed by the European Society of Cardiology and the European Society of Anaesthesiology (ESC/ESA) [17]. CAD and chronic heart failure at baseline were diagnosed according to previously described criteria. [4, 18] Cardiac consultation was performed in patients with PMI detected during routine PMI-screening, a 12-lead ECG was obtained, as well as cardiac imaging as indicated clinically.

### Hs-cTnI and hs-cTnT

Hs-cTnI (ARCHITECT High Sensitive STAT Troponin I assay, Abbott Laboratories, Illinois, USA; 99th percentile upper reference limit (URL) 26 ng/L) [19] and hs-cTnT (Elecsys, Roche Diagnostics, Mannheim, Germany; URL 14 ng/L) [4, 5] concentrations were measured before surgery and on postoperative days 1 and 2 [6, 7, 12].

### Definition of PMI, PMI_Infarct_, PMI_Injury_, and MINS

To comply with the concepts reinforced by the 4^th^ Universal Definition of Myocardial Infarction (UDMI) [12], PMI was defined as a composite of perioperative myocardial infarction (PMI_Infarct_) and perioperative myocardial injury (PMI_Injury_).

PMI_Infarct_ was defined as a rise and fall of hs-cTnI/T concentrations, which occurred in the first 3 days after surgery (during the screening period), and which was accompanied by clinical evidence of acute myocardial ischemia demonstrated by one or more of the following: ischemic ECG changes, ischemic symptoms (e.g. chest pain), new regional wall motion abnormalities, or documentation of coronary thrombus [12]. As there is no established delta for the rise and fall in the perioperative period, and to definitely fulfill the request of the UDMI of having at least one value above the URL, an absolute delta of the respective URL of each hs-cTnI/T assay (14 ng/L for hs-cTnT and 26 ng/L for hs-cTnI) above preoperative concentration (or between two postoperative concentrations if the preoperative value was missing) [4] was used. PMI_Injury_ was diagnosed if the hs-cTnI/T delta criteria for PMI_Infarct_ were met, but none of the clinical, ECG, and imaging criteria [12, 20].

MINS using hs-cTnT was defined as a postoperative hs-cTnT concentration of 20 to < 65 ng/L with an absolute change of at least 5 ng/L or hs-cTnT concentration ≥ 65 ng/L [5, 10, 11]. All patients with elevations adjudicated to be not due to ischemia such as sepsis, pulmonary embolism, heart trauma, stroke, and atrial fibrillation were not considered to have MINS. We used the same definition for hs-cTnI, except that the range of the hs-cTnI postoperative concentration was considered between 26 and < 65 ng/L (to comply with the universal definition of having a concentration above the 99th percentile URL of the assay) with an absolute change of at least 5 ng/L or hs-cTnI concentration ≥ 65 ng/L [5, 10, 11].

### Clinical endpoints and follow-up

All-cause mortality was the primary prognostic endpoint. The secondary prognostic endpoint was MACE, defined as a composite of cardiovascular death, acute MI (AMI) after day 3, acute heart failure (AHF) and clinically significant arrhythmias [12]. All-cause mortality and MACE were assessed at 30-days and at 1-year. Detailed definitions are described in Supplemental methods.

Patients were followed by outpatient clinic consultations, by phone, or by contacting their primary care physician. Additionally, the study investigators requested reports from the general practitioners, treating facilities or death registries. Patients lost to follow-up were censored at last contact.

### Statistical analysis

The incidence of overall PMI and its components (PMI_Infarct_ and PMI_Injury_) as well as MINS using hs-cTnI and hs-cTnT were calculated with 95% confidence intervals (95%CI). Overall PMI was stratified by surgical disciplines and ESC/ESA surgical risk. Comparison between baseline characteristics in patients with and without PMI diagnosed by hs-cTnI and hs-cTnT were performed using chi-square test for categorical variables and Kruskal–Wallis test for continuous variables. We calculated the incidence of mortality and MACE in patients with and without PMI with 95% CI using Kaplan–Meier estimates.

### Association of PMI with the outcomes

After evaluation of Schoenfeld residuals, we calculated multivariable adjusted hazard ratios (aHR) via Cox proportional hazards models for PMI diagnosed by hs-cTnI and by hs-cTnT for death and MACE. Based on the number of events and the consensus of requiring 10 events for each independent variable, we addressed the following predefined variables: age, RCRI score, urgent/emergency surgery, and postoperative sepsis, pneumonia and stroke [4]. To compare the prognostic impact of PMI diagnosed by hs-cTnI with hs-cTnT, the statistical significance of the difference between the aHR of PMI_Infarct_ and PMI_Injury_ diagnosed by hs-cTnI and hs-cTnT was assessed by bootstrapping. Missing data are indicated in or below the respective tables and figures. No imputation was performed for missing values. We stratified patients according to PMI_Infarct_ and PMI_Injury_ status and constructed Kaplan–Meier plots for 30-day and 1-year mortality and MACE. Curves were compared by the log-rank test. Additionally, we stratified the patients with PMI diagnosed using hs-cTnI in tertiles according to the maximum hs-cTnI delta concentration and constructed Kaplan–Meier plots for 1-year mortality and MACE for comparing the prognosis of patients with delta values in the higher tertile with the ones in the two lower tertiles.

### Sensitivity analysis

As recent studies have suggested that the approved 99th percentile of hs-cTnI (26 ng/L) may not be biologically equivalent of the 99th percentile of hs-cTnT (14 ng/L), we performed sensitivity analysis using 8.7 ng/L (biologically equivalent 99th percentile) and 16 ng/L (recently determined to be a reasonable alternative 99th percentile concentration) as the 99th percentile URL and as a delta to diagnose PMI [21, 22]. Additionally, we performed the same analysis, using 16 ng/L as an alternative 99th percentile and as a delta to diagnose PMI using hs-cTnT [21]. For these analyes, PMI overall was not stratified into PMIInfarct and PMIInjury because clinical symptoms and ECGs were not systematically done in patients with an hs-cTnI delta lower than 26 ng/L. Finally, we compared the incidence of PMI using hs-cTnT and hs-cTnI with the above-mentioned 99th percentile URL values.

As parallel hs-cTnI and hs-cTnT measurements used for the main analysis were available only in 3,111 cases of the 11,308 patients in our cohort, we performed additional analysis for all patients for whom at least one assay was available. (Supplement eFigure 1).

Statistical analyses were done using SPSS v. 24 and R v.3.6.

## Results

A total of 2,455 patients undergoing 3,111 surgical interventions were eligible for the main analysis (Supplement eFigure 1). Median patient age was 73 years, and 44% were women (Table 1). Baseline characteristics of patients with and without PMI are shown in Table 1 for hs-cTnI, Supplemental eTable 2 for PMI_Infarct_ and PMI_Injury_, and Supplemental eTable 3 for hs-cTnT.

**Table 1:** Baseline characteristics of the patients with without PMI, diagnosed by high-sensitivity cardiac troponin I (hs-cTnI)

	All surgeries	PMI_hs-cTnI_	No PMI	*P* value
	n = 3,111	n = 273	n = 2,838	
Male gender, *n* (%)	1,755 (56)	157 (58)	1,598 (56)	0.749
Age (years), median (IQR)	73 [68–79]	77 [70–81]	73 [68–78]	** < 0.001**
Diabetes mellitus, *n* (%)	760 (24)	77 (28)	683 (24)	0.186
No insulin, *n* (%)	492 (16)	46 (17)	446 (16)	
Insulin, *n* (%)	268 (9)	31 (11)	237 (8)	
Hypertension, *n* (%)	2,072 (67)	207 (76)	1,865 (66)	**0.001**
Coronary artery disease, *n* (%)	886 (29)	123 (45)	763 (27)	** < 0.001**
Peripheral artery disease, *n* (%)	568 (18)	94 (34)	474 (17)	** < 0.001**
Chronic heart failure, *n* (%)	299 (10)	57 (21)	242 (9)	** < 0.001**
Atrial fibrillation, *n* (%)	496 (16)	61 (222)	435 (15)	**0.003**
Stroke/TIA, *n* (%)	309 (10)	42 (15)	267 (9)	**0.002**
COPD^b^, *n* (%)	456 (15)	33 (12)	423 (15)	0.212
Renal dysfunction^a^, *n* (%)	1,473 (47)	152 (56)	1,321 (47)	**0.004**
Urgent/emergency Surgery, *n* (%)	690 (22)	72 (26)	618 (22)	0.093
Revised Cardiac Risk Index				
I	1,385 (45)	64 (23)	1,321 (47)	** < 0.001**
II	1,046 (34)	91 (33)	955 (34)	
III	460 (15)	76 (28)	384 (14)	
IV	220 (7)	42 (15)	178 (6)	
Preoperative Medications				
ASA, *n* (%)	1,014 (33)	128 (47)	886 (31)	** < 0.001**
Clopidogrel, *n* (%)	90 (3)	10 (4)	80 (3)	0.446
Statins, *n* (%)	1,324 (43)	146 (53)	1,178 (42)	** < 0.001**
Beta-blockers, *n* (%)	1,164 (37)	129 (47)	1,035 (37)	**0.001**
ACEI/ ARB, *n* (%)	1,489 (48)	141 (52)	1,348 (48)	0.205
Laboratory assessment				
Creatinine^c^ (mg/dL), median [IQR]	0.92 [0.75–1.17]	1.04 [0.79–1.33]	0.91 [0.75–1.15]	** < 0.001**
Hemoglobin^d^ (g/dL), median [IQR]	12.8 [11.2–14.1]	12.5 [10.9–13.9]	12.9 [11.3–14.1]	0.074

^a^Chronic kidney disease stage I–IV, ^b^*n* = 3,098 ^c^*n* = 3,066, ^d^*n* = 3,067

*TIA* transient ischemic attack, *COPD* chronic obstructive pulmonary disease, *PMI* perioperative myocardial infarction and injury, *ASA* aspirin, *ACEI* angiotensin-converting enzyme inhibitors; ARB angiotensin receptor blockers, *IQR* interquartile range

### PMI and MINS

PMI diagnosed by hs-cTnI occurred after 273 of the 3111 operations (8.8%; 95% CI 8–10%, Supplement eFigure 2). Considering the PMI individual components, PMI_Infarct_ occurred after 82 operations (2.6%; 95% CI 2.0–3.2) and PMI_Injury_ after 191 operations (6.1%; 95% CI 5.3–6.9%). MINS occurred after 344 operations (11.1%; 95% CI 10.0–12.2%). Seven percent of patients with PMI underwent coronary angiography.

PMI diagnosed by hs-cTnT occurred after 466 of the 3111 operations (15.0%; 95% CI 14–16%), PMI_Infarct_ after 116 operations (3.7%; 95% CI 3.0–4.4) and PMI_Injury_ after 350 operations (11.3%; 95% CI 10.2–12.4%). MINS occurred after 782 operations (25.1%; 95% CI 23.6–26.6). Only 4% of patients with overall PMI had chest pain, regardless of the assay used for diagnosis, and 87% had no cardiovascular symptoms. Supplemental eTable 4a and b shows the surgical characteristics and incidence of overall PMI, diagnosed by hs-cTnI and hs-cTnT, according to the type of surgery and the ESC/ESA risk classification.

### Mortality and MACE associated with PMI

**Fig. 1:**
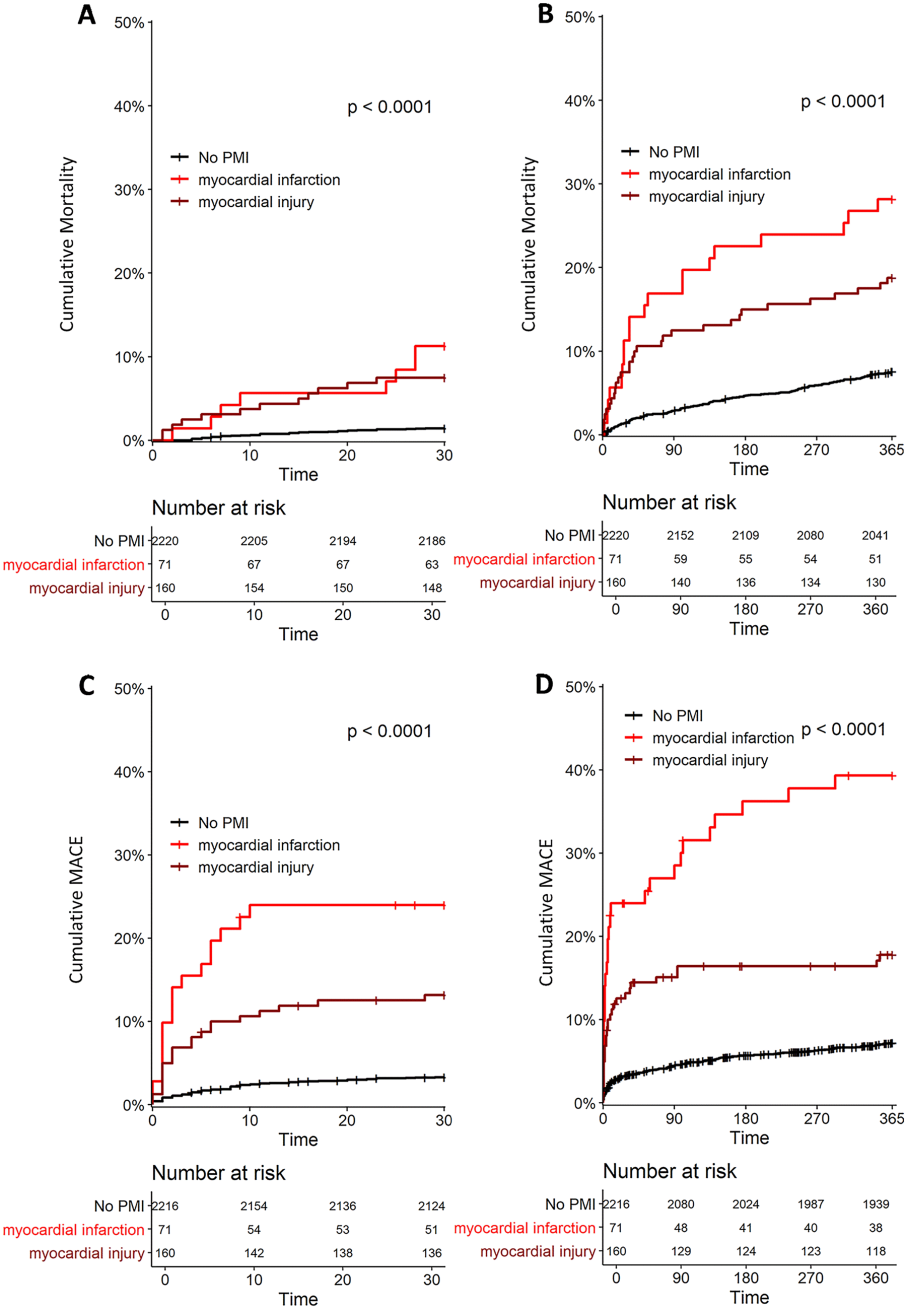
Thirty-day and 1-year mortality after surgery (Panels A and B) and MACE (Panels C and D) in patients with and without PMI diagnosed by hs-cTnI. Hs-cTnI = high-sensitivity cardiac troponin I; MACE = major adverse cardiac events; PMI = perioperative myocardial infarction and injury

**Fig. 2:**
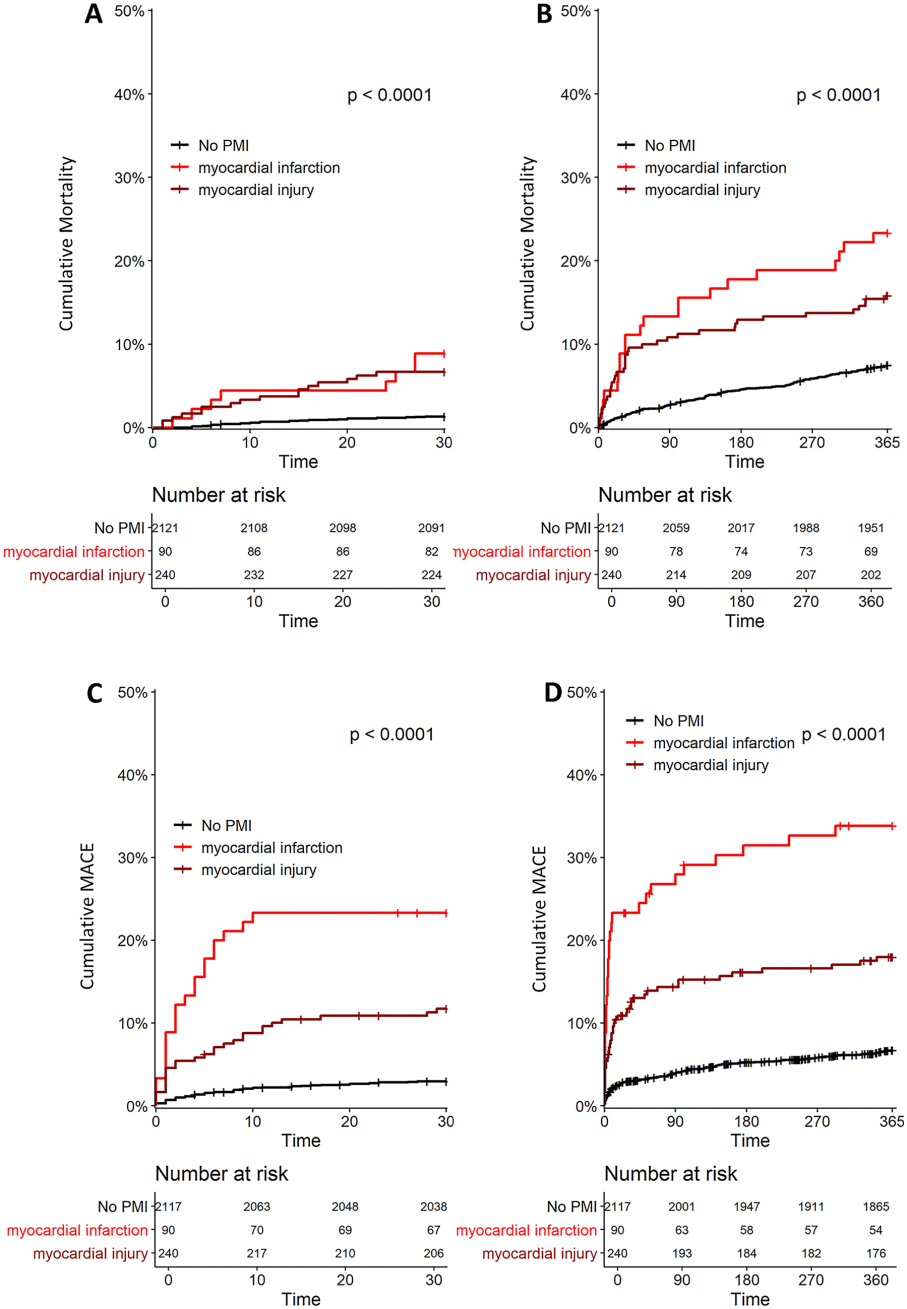
Thirty-day and 1-year mortality after surgery (Panels A and B) and MACE (Panels C and D) in patients with and without PMI diagnosed by hs-cTnT. Hs-cTnT = high-sensitivity cardiac troponin T; MACE = major adverse cardiac events; PMI = perioperative myocardial infarction and injury

Among 2455 patients eligible for this analysis, follow-up was complete in 99.8% for mortality and 99.5% for MACE. All-cause mortality occurred in 52 (2%) patients within 30 days and 217 (9%) within 1-year (Table 2). Mortality within 30 days and 1 year was significantly higher in patients with PMI versus those without (9% vs. 1% [HR 6.2, 95% CI 4–11] and 22% vs. 8% [HR 3.2, 95% CI 2–4], respectively, for hs-cTnI (Table 2, Fig. 1), *p* < 0.001; 7% vs. 1% [HR 5.7, 95% CI 3–10] and 18% vs. 7% [HR 2.6, 95% CI 2–4], respectively, for hs-cTnT (Table 2, Fig. 2], *p* < 0.001). PMI_Infarct_ and PMI_Injury_ diagnosed by hs-cTnI were independent predictors of mortality after 30 days and 1 year (Table 3), and of comparable prognostic impact versus PMI_Infarct_ and PMI_Injury_ diagnosed using hs-cTnT (*p* > 0.05; Table 4).

**Table 2:** Mortality and MACE within 30 days and 1-year after surgery in patients with or without overall PMI diagnosed by hs-cTnI and hs-cTnT

		All patients *n* = 2455 *n* (%, 95% CI)	PMI_hs-cTnI_ *n* = 231 *n* (%, 95% CI)	No PMI_hs-cTnI_ *n* = 2224 *n* (%; 95% CI)	PMI_hs-cTnT_ *n* = 330 *n* (%, 95% CI)	No PMI_hs-cTnT_ *n* = 2,125 *n* (%; 95% CI)
**Total mortality 30 days**		52 (**2%**, 1.5–3)	20 (**9%**, 5–12)	32 (**1%**, 0.9–2)	24 (**7%**, 4–10)	28 (**1%**, 0.8–1.8)
**MACE 30 days**		111 (**5%**, 4–5)	38 (**16%**, 12–21)	73 (**3%**, 2.6–4)	49 (**15%**, 11–19)	62 (**3%**, 2.2–3.7)
Cardiovascular death		24 (**1%**, 0.6–1.4)	14 (**6%,** 3–9)	10 (**0.5%**, 0.2–0.7)	16 (**5%**, 2.5–7)	8 (**0.4%**, 0.1–0.6)
Myocardial infarction		10 (**0.4%**, 0.2–0.7)	2 (**0.9%**, 0–2)	8 (**0.4%**, 0.1–0.6)	5 (**1.5%**, 0.2–3)	5 (**0.2%**, 0–0.5)
Acute heart failure		41 (**2%**, 1.2–2.2)	17 (**7%**, 4–11)	24 (**1%**, 0.7–1.5)	18 (**6%**, 3–8)	23 (**1%**, 0.7–1.5)
Arrhythmia		63 (**3%**, 2–3.2)	21 (**9%**, 5–13)	42 (**2%**, 1.3–2.5)	28 (**9%**, 6–12)	35 (**2%**, 1.1–2.2)
**Total mortality 1 year**		217 (**9%**, 8–10)	50 (**22%**, 17–27)	167 (**8%**, 6–9)	59 (**18%**, 14–22)	158 (**7%**, 6–9)
**MACE 1 year**		212 (**9%**, 8–10)	55 (**24%**, 19–30)	157 (**7%**, 6–8)	72 (**22%**, 18–27)	140 (**7%**, 6–8)
Cardiovascular death		59 (**2%**, 1.8–3)	24 (**10%**, 7–15)	35 (**2%**, 1.1–2.2)	25 (**8%**, 5–11)	34 (**2%**, 1.1–2.2)
Myocardial infarction		39 (**2%**, 1–2.2)	9 (**4%**, 2–7)	30 (**1%**, 0.9–2)	14 (**4%**, 2–7)	25 (**1%**, 0.8–1.7)
Acute heart failure		96 (**4%**, 3–5)	28 (**12%**, 9–18)	68 (**3%**, 2.4–3.9)	35 (**11%**, 8–15)	61 (**3%**, 2–4)
Arrhythmia		83 (**3%**, 2.7–4)	25 (**11%**, 7–16)	58 (**3%**, 2–3.3)	31 (**9%**, 6–13)	52 (**3%**, 1.8–3.2)

*PMI* perioperative myocardial infarction and injury, *MACE* major adverse cardiovascular events hs-*cTnI* high-sensitivity cardiac Troponin I, *hs-cTnT* high-sensitivity cardiac troponin T

**Table 3:** Multivariable Cox regression models for the prediction of MACE and mortality within 30 days and 1 year after surgery (PMI diagnosed by hs-cTnI)

		Adjusted hazard ratio (95% CI) 30 days	P value	Adjusted hazard ratio (95% CI) 1 year	*P* value
**Mortality**					
Age, per year		1.03 (0.99–1.07)	0.103	1.05 (1.02–1.07)	< 0.001
PMI					
PMI_Infarct_		**2.50 (1.05–5.96)**	**0.039**	**2.02 (1.23–3.31)**	**0.006**
PMI_Injury_		**2.79 (1.40–5.55)**	**0.004**	**1.79 (1.20–2.68)**	**0.004**
RCRI Score ≥ II		3.73 (2.10–6.61)	< 0.001	2.17 (1.63–2.89)	< 0.001
Sepsis		9.59 (4.69–19.60)	< 0.001	6.06 (3.68–10.0)	< 0.001
Pneumonia		1.67 (0.67–4.14)	0.2681	2.34 (1.41–3.89)	0.001
Stroke		3.53 (0.99–12.60)	0.053	4.66 (2.13–10.2)	< 0.001
Urgency or emergency surgery		3.21 (1.81–5.69)	< 0.001	1.47 (1.1–1.97)	0.010
**MACE**					
Age, per year		1.02 (0.99–1.04)	0.194	1.03 (1.01–1.05)	0.003
PMI					
PMI_Infarct_		**3.19 (1.78–5.73)**	** < 0.001**	**3.15 (2.02–4.92)**	** < 0.001**
PMI_Injury_		**2.22 (1.32–3.77)**	**0.003**	**1.67 (1.09–2.54)**	**0.018**
RCRI score ≥ II		3.30 (2.23–4.89)	< 0.001	2.98 (2.24–3.95)	< 0.001
Sepsis		5.53 (2.97–10.31)	< 0.001	5.41 (3.15–9.30)	< 0.001
Pneumonia		3.27 (1.79–5.98)	< 0.001	3.22 (1.97–5.28)	< 0.001
Stroke		4.35 (1.69–11.21)	0.002	3.60 (1.54–8.38)	0.003
Urgent or emergency surgery		1.95 (1.31–2.91)	0.001	1.68 (1.25–2.26)	< 0.001

*MACE* Major adverse cardiovascular events, *RCRI* Revised Cardiac Risk Index, *PMI* perioperative myocardial infarction and injury, *CI* confidence interval

**Table 4:** Multivariable Cox regression models for the prediction of MACE and mortality within 30 days and 1-year after surgery (PMI diagnosed by hs-cTnT)

		Adjusted Hazard Ratio (95%CI) 30 days	*P* value	Adjusted Hazard Ratio (95%CI) 1 year	*P* value
**Mortality**					
Age, per year		1.04 (0.99–1.08)	0.074	1.05 (1.03–1.07)	< 0.001
PMI PMI_Infarct_ PMI_Injury_		**2.32 (0.96–5.61)** **2.53 (1.30–4.91)**	**0.061** **0.006**	**1.66 (1.02–2.71)** **1.34 (0.91–1.96)**	**0.042** **0.135**
RCRI Score ≥ II		3.76 (2.11–6.68)	< 0.001	2.22 (1.67–2.96)	< 0.001
Sepsis		9.30 (4.47–19.37)	< 0.001	5.99 (3.60–9.97)	< 0.001
Pneumonia		1.38 (0.53–3.62)	0.513	2.37 (1.41–3.99)	0.001
Stroke		3.80 (1.06–13.62)	0.040	4.92 (2.24–10.80)	< 0.001
Urgency or emergency surgery		3.11 (1.74–5.53)	< 0.001	1.48 (1.10–1.99)	0.009
**MACE**					
Age, per year		1.02 (0.99–1.04)	0.194	1.03 (1.01–1.05)	0.003
PMI					
PMI_Infarct_		**3.91 (2.26–6.77)**	** < 0.001**	**3.12 (2.03–4.79)**	** < 0.001**
PMI_Injury_		**2.36 (1.47–3.79)**	** < 0.001**	**1.84 (1.28–2.64)**	**0.001**
RCRI Score ≥ II		3.18 (2.15–4.72)	< 0.001	2.95 (2.23–3.92)	< 0.001
Sepsis		5.22 (2.81–9.71)	< 0.001	5.34 (3.11–9.18)	< 0.001
Pneumonia		3.21 (1.76–5.83)	< 0.001	3.10 (1.89–5.09)	< 0.001
Stroke		3.86 (1.49–9.96)	0.005	3.33 (1.42–7.81)	0.006
Urgent or emergency surgery		2.00 (1.34–2.98)	0.001	1.69 (1.26–2.27)	< 0.001

*MACE* Major adverse cardiovascular events, *RCRI* Revised Cardiac Risk Index, *PMI* perioperative myocardial infarction and injury, *CI* confidence interval

MACE occurred in 111 (5%) patients within 30 days and in 212 (9%) within 1 year (Table 2), and was more prevalent in patients with PMI versus patients without PMI (16% vs. 3% [HR 5.4, 95% CI 4–8] and 24% vs. 7% [HR 3.9, 95% CI 3–5], respectively, for hs-cTnI (Table 2, Fig. 1), *p* < 0.001 and 15% vs. 3% [HR 5.4 95% CI 4–8] and 22% vs. 7% [HR 3.8, 95% CI 3–5], respectively, for hs-cTnT (Table 2, Fig. 2), *p* < 0.001). Additionally, PMI_Infarct_ and PMI_Injury_ diagnosed by hs-cTnI, as well as diagnosed by hs-cTnT were also independent predictors of MACE within 30 days and 1 year (Tables 3, 4). Overall, the prognostic impact of PMI_Infarct_ and PMI_Injury_ for MACE diagnosed by hs-cTnI was comparable to PMI using hs-cTnT (*p* > 0.1).

Patients with PMI and a hs-cTnI delta value in the highest tertile had worse prognosis than PMI patients with lower hs-cTnI delta concentrations (Supplemental eFigure 4).

### Sensitivity analysis

Sensitivity analysis using hs-cTnI cut-off values of 8.7 ng/L and 16 ng/L for the diagnosis of PMI showed an incidence of 15.7% (14–17%) and 11.6% (95% CI 11–13%), respectively. Using a cut-off of 16 ng/L, mortality within 30-days and 1-year was significantly higher in patients with PMI versus those without (8% vs. 1% [HR 6.3, 95% CI 4–11] and 20% vs. 7% [HR 2.9, 95% CI 2–4], respectively). MACE rates in 30-days and 1-year were also higher in patients with PMI (15% vs. 3% [HR 5.0, 95% CI 3–7] and 23% vs. 7% [HR 3.8, 95% CI 3–5], respectively). In the multivariable analysis, PMI diagnosed by a delta of 16 ng/L was also an independent predictor for death within 30 days and 1 year (aHR 3.0 [95% CI 1.7–5.4; *p* < 0.001] and aHR 1.8 [95% CI 1.3–2.5; *p* < 0.001], respectively) and MACE (aHR 2.5 [95% CI 1.7–3.9; *p* < 0.001] and aHR 2.2 [95% CI 1.6–3.1; *p* < 0.001], respectively). Regarding hs-cTnT, sensitivity analysis using a delta hs-cTnT value of 16 ng/L for the diagnosis of PMI showed an incidence of 12.1% (95% CI 11–13%). In the multivariable analysis, PMI diagnosed by hs-cTnT using a delta of 16 ng/L was also an independent predictor for death within 30 days and 1 year (aHR 2.6 [95% CI 1.4–4.7; *p*= 0.001] and aHR 1.8 [95% CI 1.3–2.4; *p* = 0.001], respectively) and MACE (aHR 2.0 [95% CI 1.3–3.1; *p* = 0.002] and aHR 2.0 [95% CI 1.4–2.7; *p* < 0.001], respectively).

The incidence of PMI diagnosed by several 99th percentile URL is shown in Supplement eTable 5.

Sensitivity analysis for the association between PMI and mortality/MACE in all patients for whom each assay was available (4,842 cases for hs-cTnI and 8,659 for hs-cTnT) is shown in Supplemental results. MINS diagnosed by hs-cTnI was an independent predictor for MACE and mortality after 30 days and 1 year (Supplemental eTable 8, Supplemental eFigure 3).

## Discussion

This prospective diagnostic multicenter study evaluated the incidence and outcome of PMI and MINS after non-cardiac surgery diagnosed by hs-cTnI, the most widely used biomarker of cardiomyocyte injury, and compared it to PMI and MINS diagnosed by hs-cTnT. Important pathophysiological and analytical differences between cTnI and cTnT mandated this analysis to complement recent studies using hs-cTnT [4, 5, 23]. We report four major findings.

First, using hs-cTnI 9% of patients developed PMI following non-cardiac surgery. Thus, the incidence of PMI using hs-cTnI was considerably lower compared to that using hs-cTnT (15%) [4, 5]. This finding was confirmed when analyzing MINS, as well as in sensitivity analysis with an even larger sample of patients, for whom each assay was available (PMI in 9% for hs-cTnI versus 16% for hs-cTnT). This difference persisted in part in sensitivity analyses using a recently suggested lower 99th percentile for hs-cTnI (16 ng/L) compared to that of hs-cTnT using the manufacturer 99^th^ percentile of 14 ng/L (11.6% for hs-cTnI and 15% for hs-cTnT) [21]. However, after comparison of this lower hs-cTnI 99^th^ percentile with an alternative 99th percentile for hs-cTnT of 16 ng/L, the difference did not persist (PMI in 11.6% for hs-cTnI and 12.1% for hs-cTnT). Additionally, by lowering the hs-cTnI 99^th^ percentile to 8.7 ng/L, there was also no difference as compared to the hs-cTnT manufacturer’s 99th percentile of 14 ng/L (PMI incidence of 15.7% for hs-cTnI and 15% for hs-cTnT). Therefore, non-biological equivalence of the approved URL of each assay may have contributed to the lower incidence observed with hs-cTnI, but a different release pattern after perioperative triggers might contribute as well. [13, 14, 24–29] Second, as described previously for PMI and MINS diagnosed with hs-cTnT, the vast majority of PMI and MINS diagnosed with hs-cTnI was asymptomatic and would have been missed without systematic screening [4, 5, 9–11, 23]. Third, the 30-day and 1-year mortality of patients developing PMI, PMI_Infarct_ and PMI_Injury_ was much higher, than those of patients without PMI, regardless of the hs-cTn assay used. Similarly, the 30-day and 1-year rate of developing MACE including spontaneous AMI, AHF, clinically relevant arrhythmias and cardiac death was fivefold and threefold higher, respectively, in patients with PMI, compared to patients without PMI, regardless of the hs-cTn assay used. All these associations persisted after multivariate adjustments. Therefore, the findings of this study provide further support to the current recommendation of the European Society of Cardiology, the American Heart Association, and the American College of Cardiology to screen high-risk patients on a regular basis [12]. There is no evidence supporting a preference for one of both hs-cTn assays for such routine screening. We also confirmed that MINS diagnosed by hs-cTnI has important prognostic significance [30–34]. Fourth, in patients with PMI, there was no difference in short- or long-term mortality between the subgroups of patients classified as PMI_Infarct_ and PMI_Injury_. This confirms prior findings with PMI_Infarct_ and PMI_Injury_ diagnosed using hs-cTnT. In contrast, the risk of developing MACE was further increased in patients classified as PMI_Infarct_. This possibly suggests that if additional criteria requested by the 4th UDMI including ischemic symptoms, ECG changes, or wall motion abnormalities are present, extensive cardiac workup including non-invasive and/or invasive coronary angiography may have the highest yield in the attempt to decrease MACE rates [9].

These findings extend and corroborate previous findings regarding PMI and MINS using hs-cTnT as important contributors to perioperative morbidity and mortality [4, 5, 23]. The substantially lower incidence of PMI and MINS using hs-cTnI as compared to hs-cTnT contributes to an increasing number of clinical differences emerging between both quantitative markers of cardiomyocyte injury [13–15, 27, 29]. The predominate triggers and the exact pathophysiological mechanisms underlying release of cTnT/I from cardiomyocytes in the perioperative setting are largely unknown and a matter of ongoing research [25, 26].

These findings are specific for the most widely validated hs-cTnI assay. Future studies are required to document the prevalence and prognostic impact of PMI and MINS diagnosed with other hs-cTnI, as well as less sensitive cTnI and cTnT assays.

**Fig. 3:**
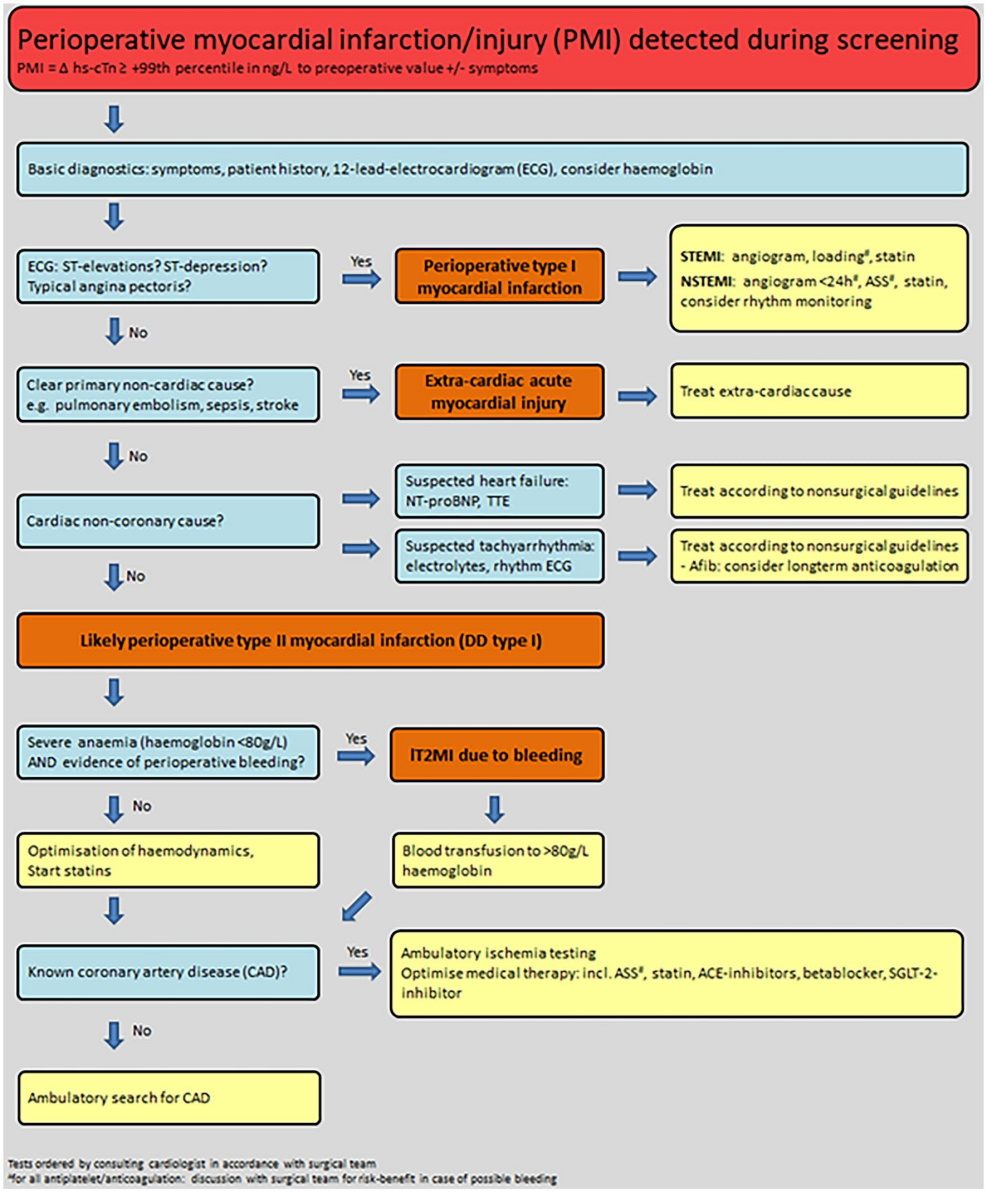
Flowchart for the management of PMIPMI = perioperative myocardial infarction and injury; STEMI = ST elevation myocardial infarction; NSTEMI = non-ST elevation myocardial infarction; afib = atrial fibrillation; TTE = echocardiogram; IT2MI = initially type 2 myocardial infarction

The most appropriate early management measures in patients detected to have PMI are only slowly evolving. Based on detailed clinical assessment including the intraoperative course combined with the 12-lead ECG and basic laboratory values including hemoglobin the most likely cause of PMI needs to be evaluated (Fig. 3). The most common etiologies include type 2 myocardial infarction, type 1 myocardial infarction, acute heart failure and tachyarrhythmia [35]. As PMI-screening by design always also provides a hs-cTnI/T concentration prior to surgery (to differentiate acute from chronic cardiomyocyte injury), and previous pilot studies had shown moderate to high prognostic accuracy of preoperative hs-cTnI/T concentration for 30-day mortality, preoperative hs-cTnI/T concentration may improve risk prediction and help physicians and patients in assessing the risk–benefit ratio of the planned surgery [18, 36].

This study has several limitations. First, screening was performed only in patients at increased cardiovascular risk. We cannot comment on the incidence and outcome of PMI or MINS in low-risk patients [37]. Second, our observations are based on the best validated and most widely used hs-cTnI assay. Further studies are necessary to evaluate to what extent our findings can be extrapolated to the recently developed novel hs-cTnI assays using other epitopes on the cTnI molecule [38, 39]. Third, as with all observational/ diagnostic studies, the strong association between PMI/MINS and the subsequent morbidity and mortality does not in itself prove causality. Fortunately, improved outcomes documented in the first randomized study targeting PMI/MINS provide hope that early management measures will allow to at least mitigate the dismal outcome of patients with PMI/MINS [40].

## Conclusions

Using hs-cTnI, PMI is less common versus using hs-cTnT, which was particularly associated with the non-biological equivalence of the approved URL of each assay. Using hs-cTnI, both PMIInfarct and PMIInjury remain independent predictors of all-cause mortality and MACE. The prognostic impact was comparable to PMIInfarct and PMIInjury diagnosed using hs-cTnT.

## Supplementary Information

Below is the link to the electronic supplementary material.

Supplementary file 1 (PDF 828 KB)

## Data Availability

DG, CP und CM had access to all the data and take responsibility for the results.
