# Supplementary material for: Incidence and outcomes of perioperative myocardial infarction/injury diagnosed by high-sensitivity cardiac troponin I
Source: Clin Res Cardiol. 2021 Mar 25;110(9):1450–63. doi: 10.1007/s00392-021-01827-w (PMC8405484; doi:10.1007/s00392-021-01827-w)

Incidence and outcomes of perioperative myocardial infarction/injury diagnosed by high sensitivity cardiac troponin I

Danielle M. Gualandro, MD, PhD*[1,2], Christian Puelacher, MD, PhD*[1], Giovanna Lurati Buse, MD[3], Noemi Glarner, MD[1], Francisco A. Cardozo,MD[2], Ronja Vogt,MD[1], Reka Hidvegi,MD[1,5], Celia Strunz,PhD[4], Daniel Bolliger,MD[5], Johanna Gueckel,MD[1], Pai C Yu,MD,PhD[2], Marcel Liffert,MD[1,5], Ketina Arslani,MD[1], Alexandra Prepoudis, MD[1], Daniela Calderaro,MD,PhD[2], Angelika Hammerer-Lercher,MD[6], Andreas Lampart,MD[5], Luzius A. Steiner, MD, PhD, Prof[7,8], Stefan Schären,MD,Prof[9], Christoph Kindler,MD,Prof[10], Lorenz Guerke, MD, Prof;[11], Stefan Osswald, MD, Prof[1], PJ Devereaux, MD,Prof[12]; Bruno Caramelli,MD,Prof[2], Christian Mueller,MD,Prof[1], for the BASEL-PMI Investigators*.

*both authors have contributed equally

[1]Department of Cardiology and Cardiovascular Research Institute Basel (CRIB) , University Hospital Basel, University of Basel, Switzerland; [2]Interdisciplinary Medicine in Cardiology Unit, Cardiology Department, Heart Institute (InCor), University of Sao Paulo Medical School, Brazil; [3]Department of Anesthesiology, University Hospital Düsseldorf, Germany; [4]Laboratory Medicine, Heart Institute (InCor), University of Sao Paulo Medical School, Brazil; [5]Department of Anesthesiology, University Hospital Basel, University of Basel, Switzerland; [6]Department of Laboratory Medicine, Cantonal Hospital Aarau, Switzerland; [7]Department of Laboratory Medicine, University of Basel, Switzerland [8]Department of Clinical Research, University of Basel, Switzerland; [9]Department of Spinal Surgery, University Hospital Basel, Switzerland; [10]Department of Anesthesiology, Cantonal Hospital Aarau, Switzerland; [11]Department of Vascular Surgery, University Hospital Basel, University of Basel, Switzerland; [12]Population Health Research Institute, David Braley Cardiac, Vascular and Stroke Research Institute, Anesthesiology, Perioperative Medicine, and Surgical Research Unit c/o Hamilton General Hospital, McMaster University, Canada.

Corresponding author: Dr. Danielle M. Gualandro

Cardiovascular Research Institute Basel (CRIB) and Department of Cardiology, University Hospital Basel, Switzerland.

Address: Spitalstrasse 2 CH-4056 Basel, Switzerland

Phone: +41 61 328 5856 Fax: +41 61 265 8577

Email:danielle.gualandro@usb.ch

Supplemental Methods

Definitions of baseline characteristics

Previous coronary artery disease (CAD) was considered in the presence of known CAD: history of myocardial infarction (MI), chronic typical exercise-induced angina pectoris, previous coronary revascularization (coronary artery bypass graft or percutaneous coronary intervention), or evidence of CAD in myocardial perfusion imaging (presence of fixed or reversible perfusion defects) or in coronary angiography.

Peripheral artery disease was defined as history of peripheral artery disease, known carotid stenosis, or arterial vascular surgery for aortic aneurysm.

Atrial fibrillation was defined as history of at least paroxysmal atrial fibrillation occurring more than once, or atrial fibrillation on preoperative electrocardiogram (ECG).

Complications

Sepsis was defined as a clinical syndrome with the presence of infection and clinical symptoms according to the International Sepsis Definitions Conference[1].

Stroke was defined as a new focal neurological deficit judged by treating physicians to be of vascular cause lasting > 24 hours.

Pneumonia was collected from the discharge diagnosis. If criteria of sepsis were fulfilled at diagnosis, sepsis was adjudicated instead.

Pulmonary embolism was collected from the discharge diagnosis.

Postoperative delirium was defined as delirium with onset within 7 days after surgery, collected from medical charts.

MACE Definitions

AMI was defined according to the criteria of the Fourth Universal Definition of Myocardial Infarction.[2] Only AMI after the screening period (after the third postoperative day) was considered as MACE. Arrhythmia (atrial fibrillation/flutter, supraventricular tachycardia, ventricular tachycardia) was considered clinically significant if requiring drug therapy or electrical cardioversion. The attending cardiologist diagnosed AHF based on clinical symptoms, physical examination, chest x-ray, B-type natriuretic peptide or N-terminal pro B-type natriuretic peptide blood concentrations, and echocardiography, in line with current heart failure guidelines.[3] Deaths were classified as cardiovascular or non-cardiovascular according to recent guidelines.[4] Deaths were assumed to be cardiovascular unless evidence of a clear non-cardiovascular cause was documented.[4]

References:

1.Levy MM, Fink MP, Marshall JC, et al. 2001 SCCM/ESICM/ACCP/ATS/SIS International Sepsis Definitions Conference. Crit Care Med 2003;31:1250–1256.

2.Thygesen K, Alpert JS, Jaffe AS, et al. Fourth universal definition of myocardial infarction (2018). Eur Heart J 2019;40:237-69.

3. Ponikowski P, Voors AA, Anker SD, et al. 2016 ESC Guidelines for the diagnosis and treatment of acute and chronic heart failure: The Task Force for the diagnosis and treatment of acute and chronic heart failure of the European Society of Cardiology (ESC) developed with the special contribution of the Heart Failure Association (HFA) of the ESC. Eur Heart J 2016;37:2129-200.

4. Hicks KA, Tcheng JE, Bozkurt B, et al. 2014 ACC/AHA Key data elements and definitions for cardiovascular endpoint events in clinical trials: a report of the American College of Cardiology/American Heart Association Task Force on clinical data standards (writing committee to develop cardiovascular endpoints data standards). Circulation 2015;132:302-61.

Supplemental Results

Sensitivity analysis using all patients for whom an individual hs-cTn assay was available

Hs-cTnI

In these analyses, 3,927 patients submitted to 4,842 surgeries were included (**eFigure 1**, **eTable 6**). The incidence of overall PMI was 8.9% (95%CI 8-10%), $PMI_{Infarct}$ was 2.6% (95%CI 2.2-3.0) and PMI_{Injury} was 6.3% (95%CI 5.6-7.0%). Hs-cTnI concentrations were above the 99th percentile (26 ng/L) in 8% of patients prior to surgery and in 16% after surgery.

Patients with overall PMI diagnosed by hs-cTnI had higher rates of mortality and MACE within 30 days (8% vs. 1% and, 15% vs. 3%, respectively) and 1 year (20% vs. 7% and, 23% vs. 7%, respectively) than patients without PMI ($P < 0.001$ for all analysis). Additionally, $PMI_{Infarct}$ and PMI_{Injury} diagnosed by hs-cTnI were independent predictors of mortality and MACE within 30 days and 1 year after surgery (**eTable 7)**.

Sensitivity analysis for MINS diagnosed by hs-cTnI

Among the 4,842 procedures included in the hs-cTnI analysis, the incidence of MINS was 12% (95%CI, 11-13%). Patients with MINS had higher all-cause mortality and MACE at 30 days (5% vs. 1.4% and 11% vs. 3%, $p<0.001$) and one year (16% vs. 8% and 20% vs. 7%, $P < 0.001$) versus patients without MINS. MINS was also an independent predictor of mortality and MACE in 30 days and 1 year. (**eTable 8, eFigure 3**)

Hs-cTnT

For these analysis, 6,965 patients submitted to 8,659 surgeries were included (**eFigure 1, eTable 9**). The incidence of overall PMI was 16% (95%CI, 15-17%), $PMI_{Infarct}$ was 3.7% (95%CI, 3.3-4.1) and PMI_{Injury} was 12.4% (95%CI, 12-13%). Hs-cTnT concentrations were above the 99th percentile (14 ng/L) in 50% of patients prior to surgery and in 63% after surgery.

Patients with overall PMI diagnosed by hs-cTnT had higher rates of mortality and MACE within 30 days (12% vs. 2% and 19% vs. 4%, respectively) and 1 year (26% vs. 9% and, 29% vs. 8%, respectively) than patients without PMI ($P < 0.001$ for all analysis). Additionally, $PMI_{Infarct}$ and PMI_{Injury} , diagnosed by hs-cTnT were independent predictors of mortality and MACE within 30 days and 1 year after surgery (**eTable 10)**.

Supplemental Tables

eTable 1. STROBE Statement—Checklist of items that should be included in reports of cohort studies.

	Item No	Recommendation	Page
**Title and Abstract**	1	(*a*) Indicate the study's design with a commonly used term in the title or the abstract.	3
		(*b*) Provide in the abstract an informative and balanced summary of what was done and what was found.	3
**Introduction**			
Background/rationale	2	Explain the scientific background and rationale for the investigation being reported.	5
Objectives	3	State specific objectives, including any prespecified hypotheses.	5-6
**Methods**			
Study design	4	Present key elements of study design early in the paper.	7
Setting	5	Describe the setting, locations, and relevant dates, including periods of recruitment, exposure, follow-up, and data collection.	7-9
Participants	6	(*a*) Give the eligibility criteria, and the sources and methods of selection of participants. Describe methods of follow-up.	7,9
		(*b*)For matched studies, give matching criteria and number of exposed and unexposed.	-
Variables	7	Clearly define all outcomes, exposures, predictors, potential confounders, and effect modifiers. Give diagnostic criteria, if applicable.	7-10 Supplement
Data sources/measurement	8*	For each variable of interest, give sources of data and details of methods of assessment (measurement). Describe comparability of assessment methods if there is more than one group.	7-9 Supplement
Bias	9	Describe any efforts to address potential sources of bias.	9-10
Study size	10	Explain how the study size was arrived at.	7-10
Quantitative variables	11	Explain how quantitative variables were handled in the analyses. If applicable, describe which groupings were chosen and why.	8-10
Statistical methods	12	(*a*) Describe all statistical methods, including those used to control for confounding.	9-10
		(*b*) Describe any methods used to examine subgroups and interactions.	10
		(*c*) Explain how missing data were addressed.	10
		(*d*) If applicable, explain how loss to follow-up was addressed.	9
		(*e*) Describe any sensitivity analyses.	10
**Results**			
Participants	13*	(a) Report numbers of individuals at each stage of study—e.g. numbers potentially eligible, examined for eligibility, confirmed eligible, included in the study, completing follow-up, and analyzed.	11 and Supplement
		(b) Give reasons for non-participation at each stage.	supplement
		(c) Consider use of a flow diagram.	supplement
Descriptive data	14*	(a) Give characteristics of study participants (e.g. demographic, clinical, social) and information on exposures and potential confounders.	24(Table 1)
		(b) Indicate number of participants with missing data for each variable of interest.	11,24 supplement
		(c) Summarise follow-up time (eg, average and total amount).	11,12
Outcome data	15*	Report numbers of outcome events or summary measures over time.	11,12, Figures 2,3

			Supplement Table 5
Main results	16	(*a*) Give unadjusted estimates and, if applicable, confounder-adjusted estimates and their precision (eg, 95% confidence interval). Make clear which confounders were adjusted for and why they were included.	11,12 Tables 3,4 supplement
		(*b*) Report category boundaries when continuous variables were categorized.	-
		(*c*) If relevant, consider translating estimates of relative risk into absolute risk for a meaningful time period.	-
Other analyses	17	Report other analyses done—e.g. analyses of subgroups and interactions, and sensitivity analyses.	12 supplement
**Discussion**			
Key results	18	Summarise key results with reference to study objectives.	13
Limitations	19	Discuss limitations of the study, taking into account sources of potential bias or imprecision. Discuss both direction and magnitude o fany potential bias.	14,15
Interpretation	20	Give a cautious overall interpretation of results considering objectives, limitations, multiplicity of analyses, results from similar studies, and other relevant evidence.	13,14
Generalizability	21	Discuss the generalizability (external validity) of the study results.	14,15
**Other information**			
Funding	22	Give the source of funding and the role of the funders for the present study and, if applicable, for the original study on which the present article is based.	1,2

eTable 2. Baseline characteristics of patients with $PMI_{Infarct}$, PMI_{Injury} and without PMI diagnosed by hs-cTnI

	**All Surgeries**	**$PMI_{hs\text{-}cTnI}$**		**No PMI**	**P**
		**$PMI_{Infarct}$**	**PMI_{Injury}**		
	n = 3,111	n=82	n = 191	n = 2,838	
Male gender, n (%)	1,755 (56)	51 (62)	106 (56)	1,598(56)	0.549
Age (years), median (IQR)	73 [68-79]	78 [69-82]	76 [70-81]	73 [68-78]	< 0.001
Diabetes mellitus, n (%)	760 (24)	30 (36)	47 (24)	683 (24)	0.064
no insulin, n(%)	492 (16)	20 (24)	26 (14)	446 (16)	
Insulin, n(%)	268 (9)	10 (12)	21 (11)	237 (8)	
Hypertension, n (%)	2,072 (67)	68 (83)	139 (73)	1,865 (66)	0.001
Coronary artery disease, n (%)	886 (29)	43 (52)	80 (42)	763 (27)	< 0.001
Peripheral artery disease, n (%)	568 (18)	40 (49)	54 (28)	474 (17)	< 0.001
Chronic heart failure, n (%)	299 (10)	23 (28)	34 (18)	242 (9)	< 0.001
Atrial fibrillation, n (%)	496 (16)	17 (21)	44 (23)	435 (15)	0.009
Stroke/TIA, n (%)	309 (10)	19 (23)	23 (12)	267 (9)	< 0.001
COPD**, n (%)	456 (15)	9 (11)	24 (13)	423 (15)	0.420
Renal dysfunction*, n (%)	1,473 (47)	44 (54)	108 (57)	1,321 (47)	0.014
Urgent/Emergency Surgery, n (%)	690 (22)	21 (26)	51 (27)	619 (22)	0.210
**Revised cardiac risk index**					
I	1,385 (45)	9 (11)	55 (29)	1,321 (47)	< 0.001
II	1,046 (34)	27 (33)	64 (34)	955 (34)	
III	460 (15)	30 (37)	46 (24)	384 (14)	
IV	220 (7)	16 (19)	26 (13)	178 (6)	
**Preoperative Medications**					
ASA, n (%)	1,014 (33)	50 (61)	78 (41)	886 (31)	< 0.001
Clopidogrel, n (%)	90 (3)	1 (1)	9 (5)	80 (3)	0.202
Statins, n (%)	1,324 (43)	54 (66)	92 (48)	1,178 (42)	< 0.001
Beta-blockers, n (%)	1,164 (37)	47 (57)	82 (43)	1,035 (37)	< 0.001
ACEI/ ARB, n (%)	1,489 (48)	53 (65)	88 (46)	1,348 (48)	0.008
**Laboratory assessment**					
**Creatinine†** (mg/dL), median [IQR]	0.92 [0.75-1.17]	1.09 [0.77-1.45]	1.02 [0.80-1.30]	0.91 [0.75-1.15]	< 0.001
Hemoglobin‡ (g/dL), median [IQR]	12.8 [11.2-14.1]	12.6 [10.5-13.6]	12.5[11.0-14.0]	12.9 [11.3-14.1]	0.079

*chronic kidney disease stage I-IV, ** n= 3,098 †n= 3,066, ‡n= 3,067; TIA= transient ischemic attack; COPD = chronic obstructive pulmonary disease; PMI= perioperative myocardial infarction and injury, ASA= aspirin; ACEI= angiotensin-converting enzyme inhibitors; ARB= angiotensin receptor blockers; IQR=interquartile range

eTable 3. Baseline characteristics of patients with and without PMI diagnosed by hs-cTnT

	**All Surgeries** n = 3,111	**PMI$_{hs\text{-}cTnT}$** n = 466	**No PMI** n = 2,645	**P -value**
Male gender, n (%)	1,755 (56)	286 (61)	1,469 (56)	0.02
Age (years), median (IQR)	73 [68-79]	76 [69-80]	73 [68-78]	< 0.001
Diabetes mellitus, n (%)	760 (24)	153 (32)	607 (23)	
no insulin, n(%)	492 (16)	90 (19)	402 (15)	< 0.001
Insulin, n(%)	268 (9)	63 (14)	205 (8)	
Hypertension, n (%)	2,072 (67)	349 (75)	1,723 (65)	< 0.001
Coronary artery disease, n (%)	886 (29)	209 (45)	677 (26)	< 0.001
Peripheral artery disease, n (%)	568 (18)	141 (30)	427 (16)	< 0.001
Chronic heart failure, n (%)	299 (10)	90 (19)	209 (8)	< 0.001
Atrial fibrillation, n (%)	496 (16)	116 (25)	380 (14)	< 0.001
Stroke/TIA, n (%)	309 (10)	64 (14)	245 (9)	0.003
COPD**, n (%)	456 (15)	87 (19)	369 (14)	0.008
Renal dysfunction*, n (%)	1,473 (47)	276 (59)	1,197 (45)	< 0.001
Urgent/Emergency Surgery, n (%)	690 (22)	130 (28)	561 (21)	0.001
**Revised cardiac risk index**				
I	1,385 (45)	109 (23)	1,276 (48)	
II	1,046 (34)	176 (38)	870 (33)	< 0.001
III	460 (15)	103 (22)	357 (14)	
IV	220 (7)	78 (17)	142 (5)	
**Preoperative Medications**				
ASA, n (%)	1,014 (33)	202 (43)	812 (31)	< 0.001
Clopidogrel, n (%)	90 (3)	19 (4)	71 (3)	0.100
Statins, n (%)	1,324 (43)	261 (56)	1,063 (40)	< 0.001
Beta-blockers, n (%)	1,164 (37)	232 (50)	932 (35)	< 0.001
ACEI/ ARB, n (%)	1,489 (48)	230 (49)	1,259 (48)	0.513
**Laboratory assessment**				
Creatinine† (mg/dL), median [IQR]	0.92 [0.75-1.17]	1.04 [0.78-1.38]	0.91 [0.75-1.13]	< 0.001
Hemoglobin‡ (g/dL), median [IQR]	12.8 [11.2-14.1]	11.1 [9.9-13.93]	13.0 [11.5-14.2]	< 0.001

*chronic kidney disease stage I-IV, **n= 3,098 †n= 3,066, ‡n= 3,067; TIA= transient ischemic attack; COPD = chronic obstructive pulmonary disease; PMI= perioperative myocardial infarction and injury, ASA= aspirin; ACEI= angiotensin-converting enzyme inhibitors; ARB= angiotensin receptor blockers; IQR=interquartile range

eTable 4A. Type of surgery and incidence of overall PMI diagnosed by hs-cTnI.

	**Incidence of PMI [95%CI]**	**ESC/ESA surgical risk**		
		**< 1%**	**1-5%**	**> 5%**
All surgeries	8.8% [7.8-9.8] (273/3,111)	4.5% [3.3-5.7] (50/1,111)	9.6% [8.2-10.1] (170/1,762)	22.3%[17.0-27.36] (53/238)
Orthopedic	7.5% [5.5-9.5] (50/667)	5.0% [2.6-7.4] (16/318)	9.6% [6.4-12.8] (31/322)	11.1% [0-22.9] (3/27)
Trauma	7.7% [4.2-11.2] (17/222)	6.4% [1.8-10.9] (7/109)	9.1% [3.7-14.4] (10/110)	0% (0/3)
**Spinal**	**9.0%** [6.7-11.3] (55/610)	0% (0/18)	9.3% [7.0-11.6] (55/592)	0% (0/0)
**Thoracic**	**12.2%** [8.2-16.1] (32/263)	0% (0/9)	10.6% [6.6-14.6] (24/227)	29.6% [12.4-46.8] (8/27)
Urologic	5% [3.2-6.8] (27/538)	4.1% [2.2-5.9] (18/438)	8.5% [2.9-14.1] (8/94)	16.7% [0-46.5] (1/6)
**Vascular**	**18.5%** [14.4-22.6] (63/340)	7.4% [4.1-14.4] (4/54)	14.1% [8.8-19.4] (23/163)	29.3% [21.4-37.2] (36/123)
Visceral	6.3% [4.0-8.6] (25/416)	3.0% [1.0-5.9] (4/133)	7.2% [3.9-10.5] (17/235)	8.3% [5.0-16.1] (4/48)
Other	7.3% [0.4-14.2] (4/55)	3.1% [0-9.1] (1/32)	10.5% [0-24.3] (2/19)	25.0% [0-74] (1/3)

PMI = perioperative myocardial infarction and injury; ESC/ESA = European Society of Cardiology/European Society of Anasthesiology; CI = confidence interval

eTable 4B. Type of surgery and incidence of overall PMI diagnosed by hs-cTnT

	**Incidence of PMI [95%CI]**	**ESC/ESA surgical risk**		
		**< 1%**	**1-5%**	**> 5%**
All surgeries	15.0% [13.7-16.3] (466/3,111)	10.2% [8.4-12.0] (113/1,111)	15.8% [14.1-17.5] (278/1,762)	31.5%[25.6-37.4] (75/238)
**Orthopedic**	**13.5%** [10.9-16.1] (90/667)	11.0% [7.6-14.4] (35/318)	15.2% [11.3-19.1] (49/322)	22.2% [6.5-37.9] (6/27)
**Trauma**	**14.9%** [10.2-19.6] (33/222)	10.1% [4.4-15.8] (11/109)	20.0% [12.7-27.3] (22/110)	0% (0/0)
**Spinal**	**14.6%** [11.8-17.4] (89/610)	33.3% [11.5-55.1] (6/18)	14.0% [11.2-16.8] (83/592)	0% (0/0)
**Thoracic**	**21.7%** [16.7-26.7] (57/263)	0% (0/9)	21.1% [15.8-26.4] (48/227)	33.3% [15.5-51.0] (9/27)
Urologic	10.0% [7.5-12.5] (54/538)	8.9% [6.2-11.6] (39/438)	14.9% [7.7-22.1] (14/94)	16.7% [0-46.5] (1/6)
**Vascular**	**27.4%** [22.7-32.1] (93/340)	20.4% [9.7-31.1] (11/54)	17.8% [11.9-23.8] (29/163)	43.1% [37.3-54.9] (53/123)
Visceral	10.3% [7.4-13.2] (43/416)	5.3% [3.4-7.2] (7/133)	12.8% [8.5-17.1] (30/235)	12.5% [3.1-21.9] (6/48)
Other	12.7% [3.9-21.5] (7/55)	12.5% [10.4-24.0] (4/32)	15.8% [0-32.2] (3/19)	0% (0/3)

ESC/ESA = European Society of Cardiology/European Society of Anasthesiology; CI = confidence interval

eTable 5. Comparison of the incidence of PMI, as diagnosed by different 99th percentile cut-offs, using hs-cTnI and hs-cTnT.

Assay	99th Percentile URL	PMI Incidence
**hs-cTnI**	8.7 ng/L	15.7% (14-17%)
	16 ng/L	11.6% (11-13%)
	26 ng/L	8.8% (8-10%)
**hs-cTnT**	14 ng/L	15% (14-16%)
	16 ng/L	12.1% (11-13%)

URL: upper reference limit; PMI: perioperative myocardial injury and infarction; hs-cTn: high-sensitivity cardiac troponin

eTable 6. Baseline characteristics of all patients with and without overall PMI diagnosed by hs-cTnI.

	**All Patients** n = 4,842	**PMI$_{hs\text{-}cTnI}$** n = 431	**No PMI** n = 4,411	**P -value**
Male gender, n (%)	2,686 (56)	238 (55)	2,448 (56)	0.919
Age (years), median (IQR)	73 [68-79]	77 [70-82]	73 [68-79]	< 0.001
Diabetes mellitus, n (%)<br>No insulin, n(%)<br>Insulin, n(%)	1,147 (24)<br>727 (15)<br>420 (9)	120 (28)<br>71 (17)<br>49 (11)	1,027 (23)<br>656 (15)<br>371 (8)	0.058
Hypertension, n (%)	3,293 (68)	340 (79)	2,953 (67)	< 0.001
Coronary artery disease, n (%)	1,325 (27)	183 (43)	1,142 (26)	< 0.001
Peripheral artery disease, n (%)	962 (20)	134 (31)	828 (19)	< 0.001
Chronic heart failure, n (%)	443 (9)	86 (20)	357 (8)	< 0.001
Atrial Fibrillation, n (%)	789 (16)	102 (24)	687 (16)	< 0.001
Stroke/TIA, n (%)	310 (6)	42 (10)	268 (6)	0.004
COPD, n (%)	754 (16)	64 (15)	690 (16)	0.678
Renal dysfunction*, n (%)	2,328 (48)	264 (61)	2,064 (47)	< 0.001
Urgent/Emergency Surgery, n (%)	1,053 (22)	116 (27)	937 (21)	0.007
**Revised Cardiac Risk Index**				
I	2,199 (45)	126 (29)	2,073 (47)	< 0.001
II	1667 (34)	143 (33)	1,524 (35)	
III	665 (14)	105 (24)	560 (13)	
IV	311 (6)	57 (13)	254 (6)	
**Preoperative Medications**				
ASA, n (%)	1,594 (33)	185 (43)	1,409 (32)	< 0.001
Clopidogrel, n (%)	140 (3)	20 (5)	120 (3)	0.026
Statins, n (%)	2,009 (42)	214 (49)	1,795 (41)	< 0.001
Beta-blockers, n (%)	1,832 (38)	201 (46)	1,631 (37)	< 0.001
ACEI/ ARB, n (%)	1,808 (37)	170 (39)	1,638 (37)	0.348
**Laboratory assessment**				
Creatinine† (mg/dL), median [IQR]	0.93 [0.76-1.18]	1.05 [0.83-1.36]	0.92 [0.75-1.16]	< 0.001
Hemoglobin† (g/dL), median [IQR]	12.7 [11.0-14.0]	12.4 [10.6-13.8]	12.7 [11.0-14.0]	0.024

*chronic kidney disease stage I-IV, † n= 4,012; ‡ n= 4,047, TIA= transient ischemic attack COPD = chronic obstructive pulmonary disease; PMI= perioperative myocardial infarction and injury, ASA= aspirin; ACEI= angiotensin-converting enzyme inhibitors; ARB= angiotensin receptor blockers; IQR = interquartile range

eTable 7. Multivariable cox regression models for the prediction of MACE and mortality within 30 days and one year after surgery (PMI diagnosed by hs-cTnI).

	**Adjusted Hazard Ratio (95%CI) 30 days**	**P -value**	**Adjusted Hazard Ratio (95%CI) one year**	**P -value**
**Mortality**				
Age, per year	1.04 (1.01-1.07)	0.022	1.05 (1.04-1.07)	< 0.001
**PMI**				
**$PMI_{Infarct}$**	**2.69 (1.27-5.72)**	**0.010**	**2.07 (1.38-3.11)**	**< 0.001**
**PMI_{Injury}**	**2.84 (1.59-5.11)**	**< 0.001**	**1.44 (1.03-2.01)**	**0.036**
RCRI Score ≥ II	3.60 (2.21-5.88)	< 0.001	2.33 (1.85-2.94)	< 0.001
Sepsis	10.06 (5.61-18.04)	< 0.001	6.01 (4.10-8.81)	< 0.001
Pneumonia	1.29 (0.55-2.98)	0.558	1.87 (1.20-2.93)	0.006
Stroke	3.94 (1.32-11.73)	0.014	4.36 (2.20-8.66)	< 0.001
Urgency or emergency surgery	2.64 (1.64-4.26)	< 0.001	1.65 (1.31-2.08)	< 0.001
**MACE**				
Age, per year	1.05 (1.02-1.07)	< 0.001	1.06 (1.04-1.07)	< 0.001
**PMI**				
**$PMI_{Infarct}$**	**3.59 (2.22-5.81)**	**< 0.001**	**3.00 (2.08-4.33)**	**< 0.001**
**PMI_{Injury}**	**1.93 (1.24-3.01)**	**0.004**	**1.59 (1.15-2.21)**	**0.005**
RCRI Score ≥ II	3.06 (2.20-4.26)	< 0.001	2.78 (2.22-3.48)	< 0.001
Sepsis	5.14 (3.19-8.28)	< 0.001	5.42 (3.67-8.02)	< 0.001
Pneumonia	3.68 (2.25-6.03)	< 0.001	3.04 (2.03-4.56)	< 0.001
Stroke	5.53 (2.47-12.36)	< 0.001	4.93 (2.47-9.84)	< 0.001
Urgent or emergency surgery	1.75 (1.26-2.43)	0.001	1.69 (1.35-2.12)	< 0.001

MACE= Major adverse cardiovascular events, RCRI= Revised Cardiac Risk Index, PMI= perioperative myocardial infarction and injury; CI = confidence interval

eTable 8. Multivariable cox logistic regression models for prediction of mortality and MACE within 30 days and one year after surgery in patients with MINS.

	**Adjusted Hazard Ratio (95%CI) 30 days**	**P -value**	**Adjusted Hazard Ratio (95%CI) one year**	**P -value**
**Mortality**				
Age, per year	1.03 (1.00-1.07)	0.040	1.05 (1.04-1.07)	< 0.001
**MINS**	**1.98 (1.16-3.39)**	**0.012**	**1.58 (1.22-2.06)**	**0.001**
RCRI Score ≥ II	3.67 (2.25-5.98)	< 0.001	2.37 (1.88-3.00)	< 0.001
Sepsis	11.7 (6.56-20.8)	< 0.001	6.48 (4.42-9.48)	< 0.001
Pneumonia	1.50 (0.65- 3.44)	0.343	1.98 (1.26-3.09)	0.003
Stroke	7.69 (2.70-21.9)	< 0.001	5.73 (2.93-11.20)	< 0.001
Urgent or emergency surgery	2.50 (1.55-4.04)	< 0.001	1.64 (1.30-2.07)	< 0.001
**MACE**				
Age, per year	1.04 (1.02-1.06)	< 0.001	1.05 (1.04-1.07)	< 0.001
**MINS**	**2.34 (1.65-3.34)**	**< 0.001**	**2.08 (1.62-2.66)**	**< 0.001**
RCRI Score ≥ II	3.15 (2.27-4.36)	< 0.001	2.81 (2.25-3.52)	< 0.001
Sepsis	5.67 (3.50-9.16)	< 0.001	6.07 (4.10-8.99)	< 0.001
Pneumonia	3.90 (2.39-6.36)	< 0.001	3.04 (2.02-4.56)	< 0.001
Stroke	11.93 (5.51-25.84)	< 0.001	8.84 (4.52-17.28)	< 0.001
Urgent or emergency surgery	1.76 (1.27-2.45)	0.001	1.69 (1.34-2.12)	< 0.001

MACE = Major adverse cardiovascular events, RCRI= Revised Cardiac Risk Index, MINS= myocardial injury after noncardiac surgery; CI = confidence interval

eTable 9. Baseline characteristics of all patients with and without overall PMI diagnosed by hs-cTnT.

	**All Patients** n = 8,659	**PMI$_{hs-cTnT}$** n = 1,392	**No PMI** n = 7,267	**P -value**
Male gender, n (%)	5,080 (59)	866 (62)	4,214 (58)	0.003
Age (years), median (IQR)	73 [68-79]	75 [69-80]	73 [60-78]	< 0.001
Diabetes mellitus, n (%)<br>No insulin, n(%)<br>Insulin, n(%)	2,239 (26)<br>1,418 (16)<br>821 (10)	433 (32)<br>230 (17)<br>203 (15)	1,736 (24)<br>1,118 (16)<br>618 (9)	< 0.001
Hypertension, n (%)	5,825 (67)	1,026 (74)	4,799 (66)	< 0.001
Coronary artery disease, n (%)	2,690 (31)	621 (45)	2,069 (29)	< 0.001
Peripheral artery disease, n (%)	2,533 (29)	542 (39)	1,991 (27)	< 0.001
Chronic heart failure, n (%)	1,077 (12)	311 (22)	766 (11)	< 0.001
Atrial Fibrillation, n (%)	1,413 (18)	336 (28)	1,077 (16)	< 0.001
Stroke/TIA, n (%)	1,007 (12)	196 (14)	811 (11)	0.002
COPD, n (%)	1,305 (15)	260 (19)	1,045 (15)	< 0.001
Renal failure*, n (%)	4,115 (48)	806 (58)	3,309 (46)	< 0.001
Urgent/Emergency Surgery, n (%)	2,389 (28)	485 (35)	1,094 (26)	< 0.001
**Revised Cardiac Risk Index**				
I	3,370 (39)	301 (22)	3,069 (42)	< 0.001
II	2,874 (33)	467 (34)	2,407 (33)	
III	1,561 (18)	345 (25)	1216 (17)	
IV	854 (10)	279 (20)	575 (8)	
**Preoperative Medications**				
ASA, n (%)	3,460 (40)	656 (47)	2,804 (39)	< 0.001
Clopidogrel, n (%)	461 (5)	83 (6)	378 (5)	0.249
Statins, n (%)	4,041 (47)	779 (56)	3,262 (45)	< 0.001
Beta-blockers, n (%)	3,438 (40)	683 (49)	2,755 (38)	< 0.001
ACEI/ ARB, n (%)	4,089 (47)	663 (48)	3,426 (47)	0.740
**Laboratory assessment**				
Creatinine† (mg/dL), median [IQR]	0.94 [0.77-1.22]	1.06 [0.80-1.48]	0.93 [0.76-1.18]	< 0.001
Hemoglobin‡(g/dL), median [IQR]	12.7 [11.0-14.1]	11.6 [9.8-13.3]	12.9 [11.3-14.2]	< 0.001

*chronic kidney disease stage I-IV, † n=8,455; ‡ n=8,463; TIA= transient ischemic attack COPD = chronic obstructive pulmonary disease; PMI= perioperative myocardial infarction and injury, ASA= aspirin; ACEI= angiotensin-converting enzyme inhibitors; ARB= angiotensin receptor blockers; IQR = interquartile range

eTable 10. Multivariable cox regression models for the prediction of MACE and mortality within 30 days and one year after surgery (PMI diagnosed by hs-cTnT).

	**Adjusted Hazard Ratio (95%CI) 30 days**	**P -value**	**Adjusted Hazard Ratio (95%CI) one year**	**P –value**
**Mortality**				
Age, per year	1.05 (1.02-1.07)	< 0.001	1.05 (1.04-1.06)	< 0.001
**PMI**				
**$PMI_{Infarct}$**	**3.45 (2.17-5.48)**	**< 0.001**	**2.21 (1.67-2.92)**	**< 0.001**
**PMI_{Injury}**	**2.68 (1.85-3.86)**	**< 0.001**	**1.84 (1.52-2.24)**	**< 0.001**
RCRI Score ≥ II	2.45 (1.80-3.35)	< 0.001	2.06 (1.76-2.41)	< 0.001
Sepsis	4.80 (3.13-7.35)	< 0.001	3.27 (2.41-4.44)	< 0.001
Pneumonia	2.24 (1.43-3.51)	< 0.001	2.19 (1.65-2.89)	< 0.001
Stroke	2.71 (1.19-6.20)	0.018	1.99 (1.14-3.46)	0.015
Urgency or emergency surgery	3.34 (2.41-4.63)	< 0.001	1.52 (1.30-1.79)	< 0.001
**MACE**				
Age, per year	1.04 (1.02-1.05)	< 0.001	1.04 (1.03-1.05)	< 0.001
**PMI**				
**$PMI_{Infarct}$**	**4.33 (3.11-6.04)**	**< 0.001**	**3.03 (2.33-3.94)**	**< 0.001**
**PMI_{Injury}**	**3.17 (2.44-4.10)**	**< 0.001**	**2.48 (2.06-2.99)**	**< 0.001**
RCRI Score ≥ II	2.08 (1.66-2.61)	< 0.001	2.38 (2.04-2.79)	< 0.001
Sepsis	3.96 (2.81-5.60)	< 0.001	3.78 (2.80-5.10)	< 0.001
Pneumonia	2.98 (2.17-4.10)	< 0.001	2.29 (1.74-3.03)	< 0.001
Stroke	4.09 (2.33-7.17)	< 0.001	2.54 (1.51-4.26)	< 0.001
Urgent or emergency surgery	1.85 (1.47-2.32)	< 0.001	1.51 (1.28-1.77)	< 0.001

MACE= Major adverse cardiovascular events, RCRI= Revised Cardiac Risk Index, PMI= perioperative myocardial infarction and injury; CI = confidence interval

Supplemental Figures

eFigure 1. Flowchart of inclusion and availability of assays

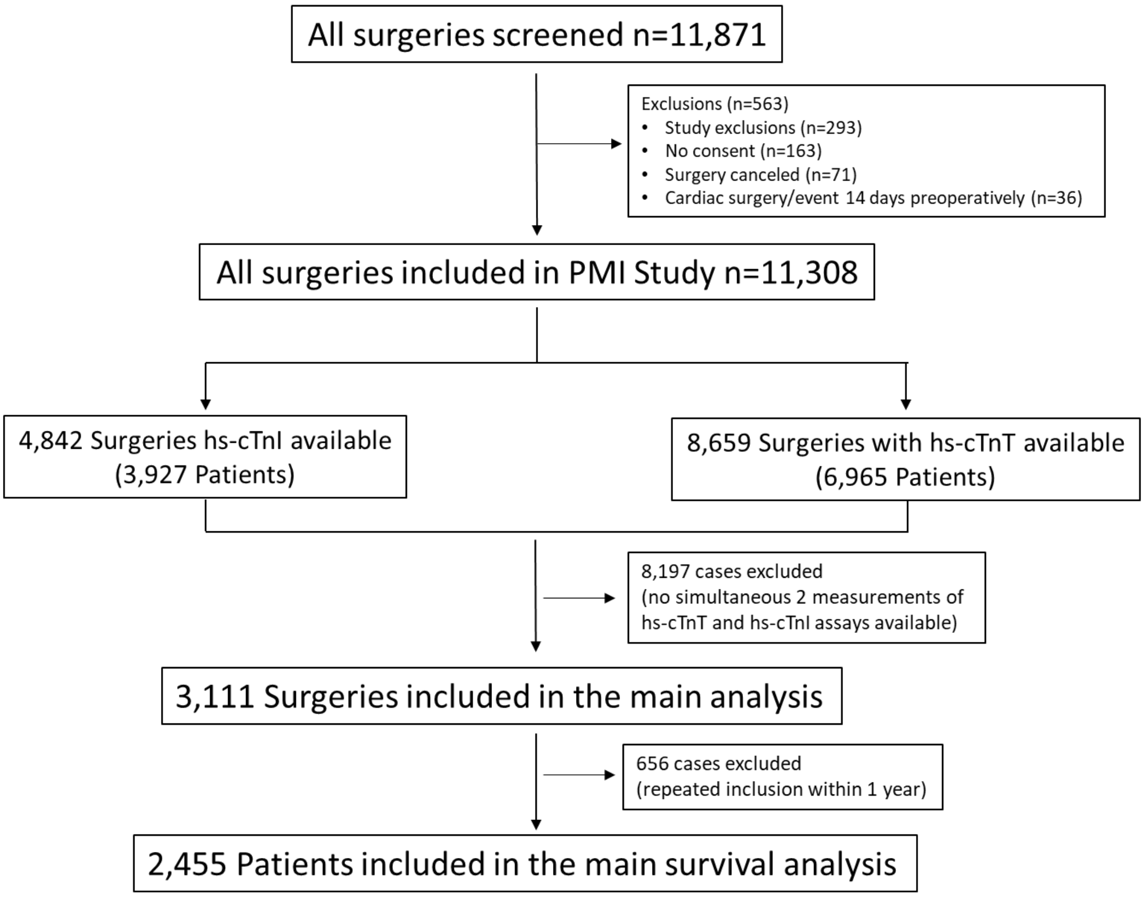

eFigure 2. Incidence of PMI and its components and MINS, quantified by hs-cTnI and hs-cTnT.

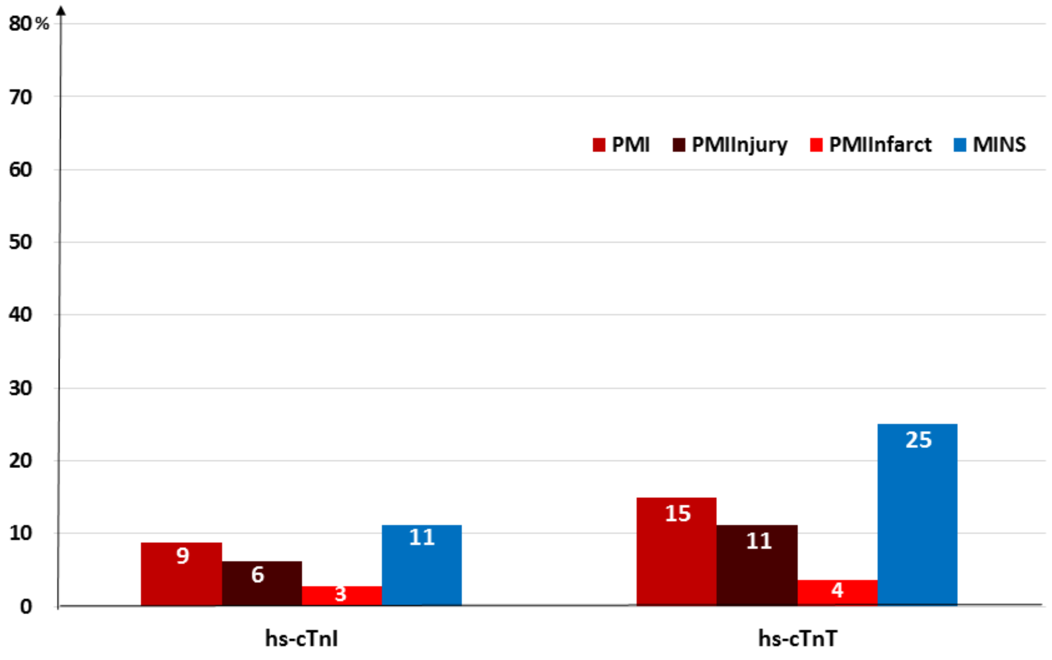

Hs-cTnI = high-sensitivity cardiac troponin I; hs-cTnT= high-sensitivity cardiac troponin T; PMI= perioperative myocardial infarction and injury; $PMI_{Infarct}$= Perioperative myocardial infarct; PMI_{Injury}= perioperative myocardial injury; MINS= myocardial injury after non-cardiac surgery.

eFigure 3. Thirty-day and 1-year mortality (Panels A and B) and MACE (Panels C and D) in patients with and without MINS diagnosed by hs-cTnI.

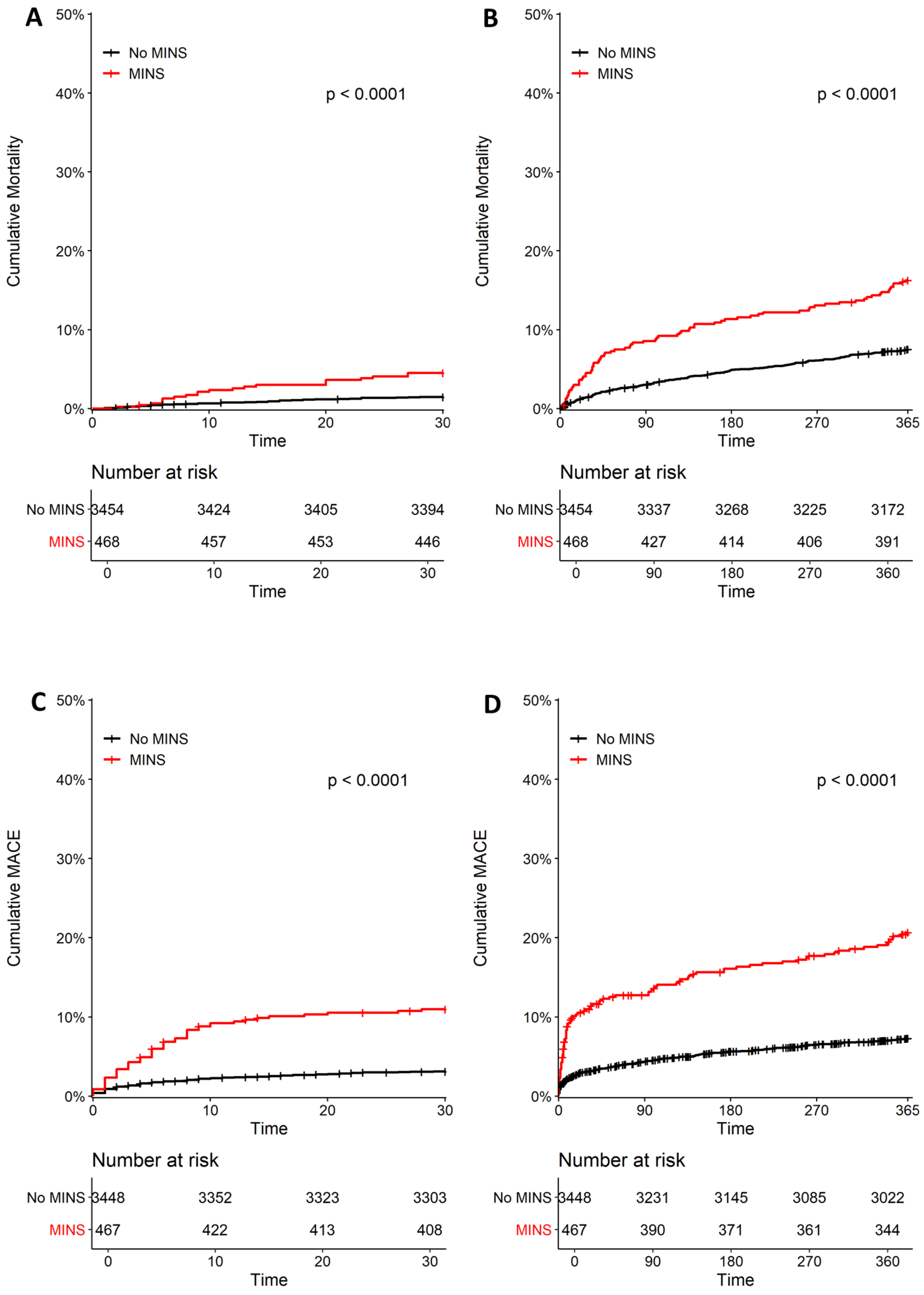

eFigure 4. One-year mortality (Panels A) and MACE (Panel B) in patients with PMI diagnosed by hs-cTnI according to the maximum hs-cTnI delta, stratified according to tertiles.

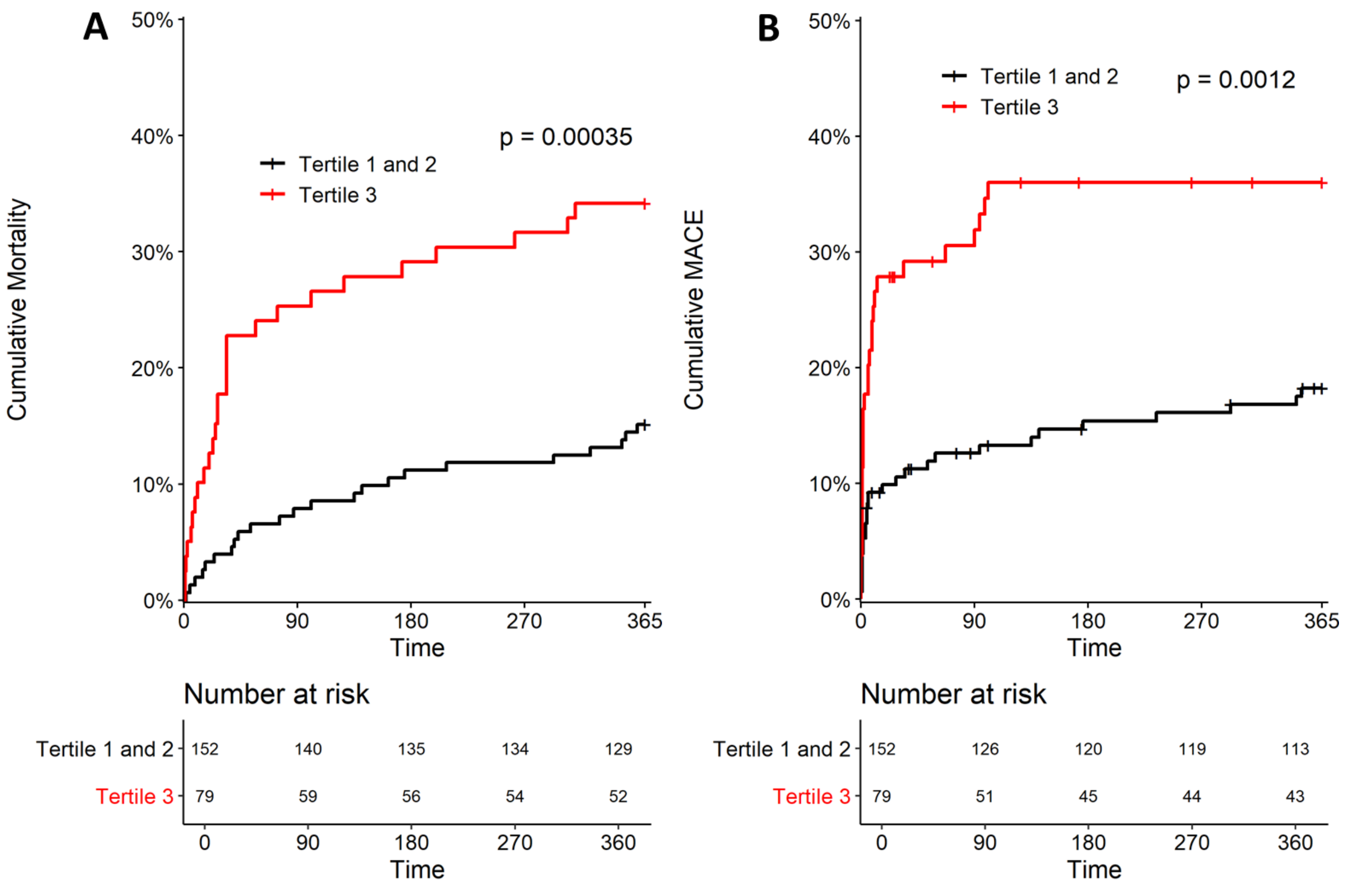

Supplement: Supplementary file 1 — (PDF 828 KB) [file 392_2021_1827_MOESM1_ESM.pdf]
